# Synthesis, Characterization and Antifungal Evaluation of 5-Substituted-4-Amino-1,2,4-Triazole-3-Thioesters

**DOI:** 10.3390/molecules16021297

**Published:** 2011-01-28

**Authors:** Aurangzeb Hasan, Noel Francis Thomas, Shelly Gapil

**Affiliations:** Department of Chemistry, Faculty of Science, University of Malaya, Kuala Lumpur-50603, Malaysia

**Keywords:** triazolethioesters, oxadiazolethioesters, oxadiazoles, triazoles, *in vitro* antifungal activity

## Abstract

A series of 5-substituted-4-amino-1,2,4-triazole-3-thioesters was synthesized by converting variously substituted organic acids successively into the corresponding esters, hydrazides, 5-substituted-1,3,4-oxadiazole-2-thiols, 5-substituted-1,2,4-triazole-2-thiols and 5-substituted-1,3,4-oxadiazole-2-thioesters. Finally the target compounds were obtained by refluxing 5-substituted-1,3,4-oxadiazole-2-thioesters in the presence of hydrazine hydrate and absolute alcohol. The structures of the synthesized compounds were established by physicochemical and spectroscopic methods. The synthesized compounds were evaluated for their *in vitro* antifungal activity. Some of the evaluated compounds possessed significant antifungal activity as compared to a terbinafine standard.

## 1. Introduction 

1,2,4-Triazoles and their derivatives constitute an important class of organic compounds with diverse agricultural, industrial and biological activities [1,2,3] including anti-microbial [4,5] sedative, anti-convulsant [6] and anti-inflammatory properties [7], and consequently, the synthesis of these heterocycles has received considerable attention in recent years [8,9,10,11,12,13,14]. In this work we report the synthesis of some new 5-substituted-4-amino-1,2,4-triazole-3-thioesters through the intermolecular cyclization of variably substituted acid hydrazides to the corresponding 5-substituted-1,3,4-oxadiazoles-2-thioles, 5-substituted-1,2,4-triazoles-2-thioles and 5-substituted-1,3,4-oxadiazoles-2-thioesters. 

## 2. Results and Discussion 

**Scheme 1:**
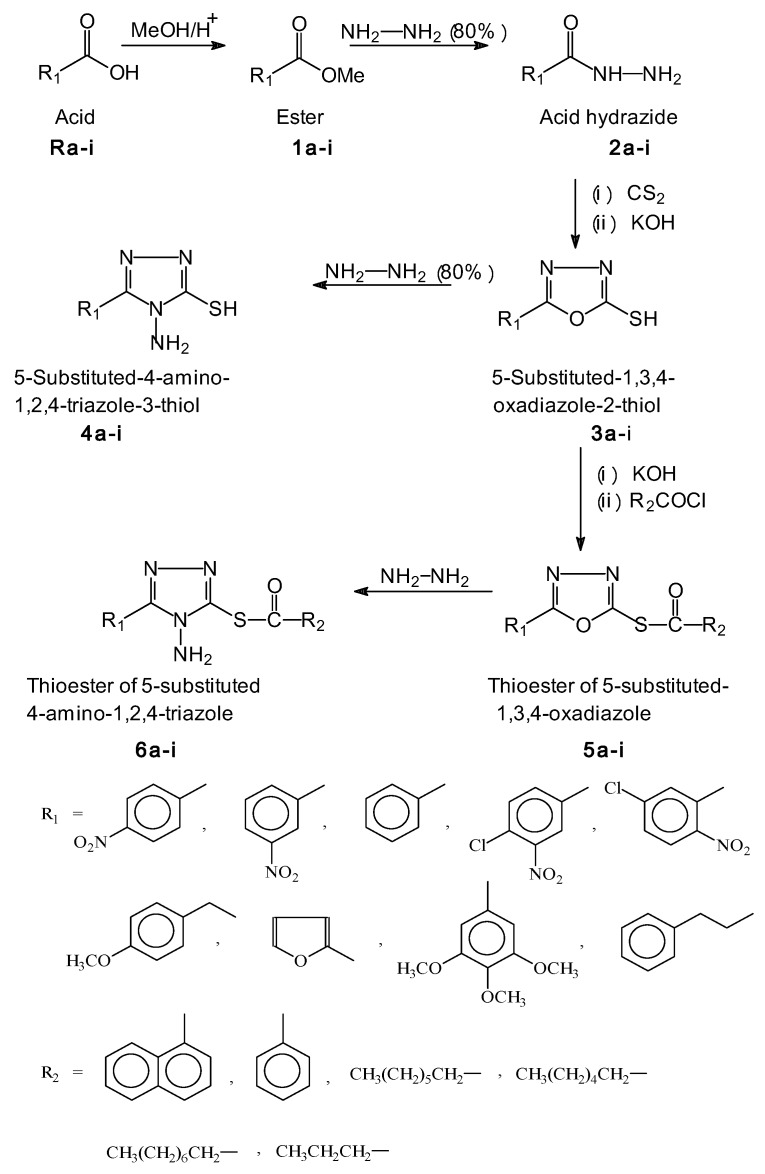
Synthesis of 5-substitued-4-amino-1,2,4-triazole-3-thioesters.

Nine 5-substituted-4-amino-1,2,4-triazole-3-thioesters were obtained in 66-83% yield by converting variously substituted organic acids **Ra-i** to the corresponding esters **1a-i** and acid hydrazides **2a-i** by reaction with methanol and hydrazine hydrate, respectively. The hydrazides were converted to 5-substituted-1,3,4-oxadiazole-2-thiols **3a-i** and 5-substituted-1,2,4-triazole-2-thiols **4a-i** as described in [15,16]. 5-Substituted-1,3,4-oxadiazole-2-thiols and 5-substituted-1,2,4-triazole-2-thiols were next converted to 5-substituted-1,3,4-oxadiazole-2-thioesters **5a-i** using potassium hydroxide and acid chlorides and finally to 5-substituted-4-amino-1,2,4-triazole-3-thioesters **6a-i** by refluxing the 5-substituted-1,3,4-oxadiazole-2-thioesters in the presence of hydrazine hydrate and absolute ethanol, as outlined in Scheme 1.

**Scheme 2:**
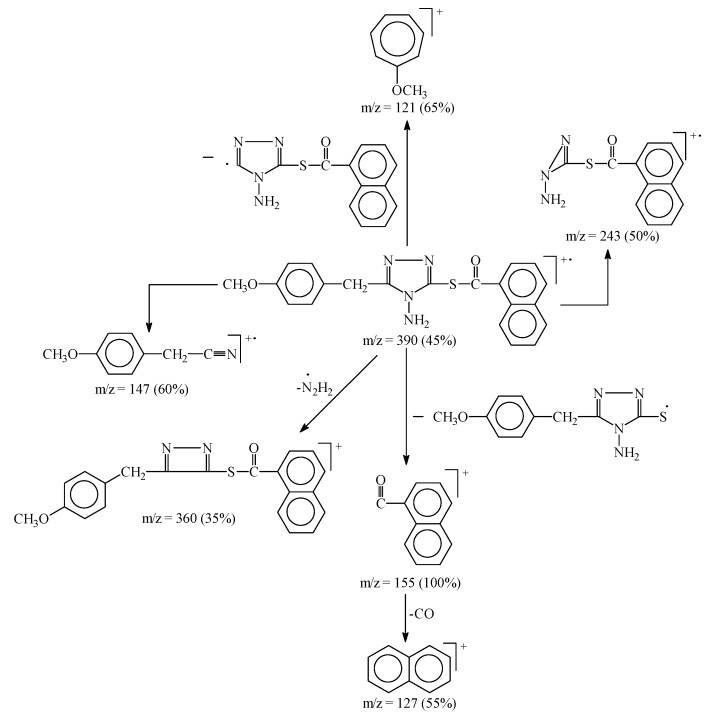
Mass fragmentation pattern of thioester **6a**.

Mass spectral data support the proposed structures. Scheme 2 shows the fragmentation pattern for 5-(4-methoxybenzyl)-4-amino-1,2,4-triazole-2-ylnapthalene-1-carbothioate (**6a**) which serves as an example for all the compounds *mutatis mutandis*. The mass spectrum showed various characteristic peaks. A peak at *m/z* 390 was assigned to the molecular ion. A peak at *m/z* 155 (the base peak) was due to the naphthoyl fragment and a characteristic peak at *m/z* 121 was attributed to the tropylium ion produced by the loss of triazole-3-thioester moiety.

The FTIR spectrum showed absorption bands at 1,518 cm^–1^, 3,204 cm^–1^, 1,684 cm^–1^ and 1,575 cm^-1^ which were assigned to C=N, N–H, C=O (ester), C–O and C=C (aromatic), respectively. The ^1^H-NMR spectrum of **6a** showed characteristic signals at 6.90 to 7.85 ppm which were assigned to the aromatic protons. A signal at 2.50 ppm was assigned to the methylene proton. The singlet at 3.56 and 3.83 ppm were assigned to the methoxy and amino protons, respectively.

**Figure 1:**
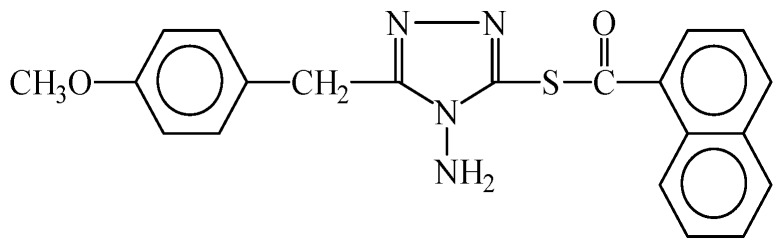
Structure of Compound (**6a**).

The ^13^C-NMR spectrum of **6a** showed characteristic peaks at 120.5-134.5 ppm which were assigned to the aromatic carbon atoms. A peak at 180.01 ppm was attributed to the carbonyl carbon atom. The peaks at 166.04 and 175.10 ppm were assigned to the carbon atoms of the triazole moiety. The peaks at 30.6 and 55.9 ppm were assigned to the methylene and methoxy carbon atoms. The significant ^13^C-NMR spectra signals are listed in Table 2 and are in agreement with the proposed structure. On the basis of the combined physical and spectral data compound **6a** was assigned the following structure, corresponding to 5-(4-methoxybenzyl)-4-amino-1,2,4-triazole-3-ylnaphthalene-1-carbothioate (Figure 1): 

Similarly, the other members of the series of 5-substituted-4-amino-1,2,4-triazole-3-thioester **6b-i** were synthesized by the ring opening reaction due to the nucleophilic attack of hydrazine on the ring carbon of the 5-substituted-1,3,4-oxadiazole-2-thioesters, followed by dehydration. The ^1^H-NMR and ^13^C-NMR, of the 5-substituted-1,3,4-oxadiazole-2-thioesters are outlined in Table 1 and Table 2 respectively.

In the *in vitro* antifungal bioassay, the compounds **6a, 6b, 6d** and **6g** were found to have significant antifungal activities against the four tested fungal strains (Table 3). In particular **6a** and **6g** had high inhibitory effects on the growth of *Aspergillus. flavus, Mucor species, Aspergillus niger* and *Aspergillus fumigatus*. A structure activity relationship comparison shows that the presence of naphthyl group at the 3-position of the triazole-3-thioester enhances the activity of the compounds. It is interesting to note that a similar group is also present in the structure of the reference compound terbinafine.

The antifungal assay results also show that the activity of the compounds **6c, 6e, 6f, 6h** and **6i** is relatively low as compared to other members of the series due to the absence of this group in these structures. It is therefore concluded that the potential pharmacophore in the triazole-3-thioesters is probably naphthalene-1-carbothioate.

## 3. Experimental 

### 3.1. General

Melting points of the synthesized compounds were recorded on Gallenkamp digital melting point apparatus MFB-595-101M in open-end capillary tubes and are uncorrected. Thin layer chromatography was carried out on pre-coated silica gel plates (0.2 mm, E. Merck, 20 × 20 cm, 60F_254_). FTIR spectra were recorded on a Bio-Red Merlin Spectrophotometer using KBr discs.^1^H-NMR (300 MHz) and ^13^C-NMR (75.43 MHz) spectra were recorded on Bruker AM-250 Spectrometer in DMSO and CDCl_3_ solutions using TMS as internal standard. EIMS were recorded on Agilent VG: 70 SE Mass Spectrometer. 

### 3.2. Procedure for the preparation of methyl-4-nitrobenzoate (***1a***)

4-Nitrobenzoic acid (5 g, 0.029 mol) was taken in a 250 mL round bottom flask fitted with a reflux condenser and a calcium chloride guard tube. Absolute methyl alcohol (25 mL) and few drops of concentrated sulphuric acid were added and the reaction mixture was refluxed for 4 hours. The reflux time was monitored by thin layer chromatography (tlc) (silica; ethyl acetate-pet. ether, 1:2). After the completion of the reaction the excess of alcohol was distilled off on a rotary evaporator. The residue was poured into 250 mL of water contained in a separating funnel. Dichloromethane (20 mL) was added to the separating funnel and the mixture was shaken vigorously. The solution was allowed to stand and the methyl-4-nitrobenzoate in the dichloromethane separated and settled at the bottom of the separating funnel. The lower layer was carefully separated and the upper aqueous layer was rejected. The methyl-4-nitrobenzoate was returned to the funnel and shaken with a strong solution of sodium bicarbonate until all the free acid was removed. Methyl-4-nitrobenzoate was washed once with water and dried by pouring into a small dry conical flask containing 2 g of anhydrous magnesium sulphate. It was shaken for 5 minutes and allowed to stand for one hour. The methyl-4-nitrobenzoate solution was filtered through a small fluted filter paper into a distillation flask. The flask was fitted with 360° thermometer, a condenser and a receiving flask. Dichloromethane was distilled off at 40 °C and the solid methyl-4-nitrobenzoate was obtained from the flask. Ethanol was used as solvent for recrystallization of the ester. 

### 3.3. Procedure for the preparation of 4-nitrobenzoic acid hydrazide (***2a***)

Methyl-4-nitrobenzoate ester (7 g, 0.041 mol) was dissolved in absolute ethanol (40 mL) and taken in a flask fitted with a reflux condenser and a calcium chloride guard tube. Hydrazine hydrate (80%, 13 mL) was added and the reaction mixture was refluxed for eight hours. The reflux time was monitored by tlc as before. After the completion of the reaction, the excess hydrazine was distilled off. The crude solid was collected, washed with water and recrystallized from 30% aqueous ethanol. 

### 3.4. Procedure for the preparation of 5-(4-nitrophenyl)-1,3,4-oxadiazole-2-thiol (***3a***)

4-Nitrobenzoic hydrazide (7 g, 0.038 mol) was dissolved in absolute ethanol (40 mL) in a 250 mL flask. Carbon disulfide (2 mL, 0.034 mol) was then added to the solution followed by the addition of a solution of potassium hydroxide (1.2 g, 0.019 mol) in water (20 mL). The reaction mixture was thoroughly stirred and refluxed. It was initially yellow which turned to green and then light yellow with the progress of the reaction. Evolution of hydrogen sulfide gas was observed during each reaction. After completion of the reaction, excess of ethanol was removed under reduced pressure. The mixture was diluted with distilled water (200 mL) and acidified with 4N hydrochloric acid to pH 2-3. It was then filtered, washed with diethyl ether and recrysallized from ethanol. 

### 3.5. Procedure for the preparation of 5-(4-nitrophenyl)-4-amino-1,2,4-triazole-3-thiol (***4a***)

5-(4-Nitrophenyl)-1,3,4-oxadiazole-2-thiol (4 g, 0.017 mol) and 80% hydrazine hydrate (6 mL, 0.124 mol) in absolute ethanol (25) were refluxed in a 250 mL flask fitted with a condenser and a guard tube. After the completion of the reaction the solvent and excess of hydrazine hydrate were removed under reduced pressure using rotary evaporator. The residue was washed with diethyl ether and recrystallized from ethanol. 

### 3.6. Procedure for the preparation of 5-(4-methoxybenzyl)-1,3,4-oxadiazole-2-ylnaphthalene-1-carbothioate (***5a***)

5-(4-Methoxybenzyl)-1,3,4-oxadiazole-2-thiol (5 g, 0.021 mol) was dissolved in absolute ethanol (30 mL) in a 250 mL flask. Potassium hydroxide (1 g, 0.178 mol) solution in water (20 mL) was added to the solution. After one hour reflux 1-naphthoyl chloride (4 g, 0.021 mol) was added to the reaction mixture. The reaction mixture was thoroughly stirred and refluxed for six hours. After completion of the reaction, the excess of ethanol in the reaction mixture was under reduced pressure. The mixture left behind was diluted with distilled water (200 mL) and then filtered. The crude product was dried in an oven and recrystallized from ethanol. 

### 3.7. Preparation for the preparation of 5-(4-methoxybenzyl)-4-amino-1,2,4-triazole-3-ylnaphthalene-1-carbothioate (***6a***). 

*5*-(4-Methoxybenzyl)-1,3,4-oxadiazole-2-ylnaphthalene-1-carbothioate (5 g, 0.013 mol) and 80% hydrazine hydrate (8 mL, 0.165 mol) in absolute ethanol (30 mL) were refluxed in a 250 mL flask fitted with a condenser and a guard tube. After completion of the reaction, the solvent and excess hydrazine hydrate were removed under reduced pressure. The residue was washed with diethyl ether and recrystallized from ethanol. 

Purification of all the synthesized compounds was achieved by recrystallization and purity of each compound was monitored by thin layer (tlc) and gas (gc) chromatography.

*5-(4-Nitrophenyl)-4-amino-1,2,4-triazole-3-ylnaphthalene-1-carbothioate* (**6a**). 5-(4-Nitrophenyl)-1,3,4-oxadiazole-2-yl naphthalene-1-carbothioate (3.0 g; 8 mmol), NH_2_-NH_2_ (5.10 g; 103 mmol) were reacted according to the general procedure. Color light brown; yield: 80% (2.40 g); recrystallization from EtOH; m.p. 168-170 °C; Molecular formula C_19_H_13_O_3_N_5_S; Mol. Wt. 191. FTIR (KBr, cm^-1^): 3250 (N-H), 1557 (C=C)_Ar_, 1633 (C=O), 1517 (C=N); ^1^H-NMR (DMSO-d_6_, δ, ppm): 7.72 (dd, 1H, *J* = 1.2, 4.6 Hz, ArH-1), 8.03 (dd, 1H, *J* = 2.5, 7.2 Hz, Ar H-2) 8.01 (dd, 1H, *J* = 2.4, 7.1 Hz, A H-3), 7.73 (dd, 1H, *J* = 2.3, 4.4 Hz, Ar H-4), 7.76 (m, 1H, Ar H-1´ ),7.42 (m, 1H, Ar H-2´ ), 7.90 (m, 1H, Ar H-3´ ), 7.88 (m, 1H, Ar H-4´ ), 7.55 (m, 1H, Ar H-5´ ), 4.32 (s, 2H, NH_2_); ^13^C-NMR (DMSO-d_6_, δ, ppm): 129.41 (C-1), 128.40 (C-2), 116.52 (C-3), 115.52 (C-4), 116.31 (C-5), 130.43 (C-6), 160.42 (C-7), 171.40 (C-8), 182.31 (C-9), 135.61 (C-10), 127.12 (C-11), 120.43 (C-12), 122.41(C-13), 125.32 (C-14), 126.32 (C-15), 128.43 (C-16), 129.42 (C-17), 130.21 (C-18), 129.66 (C-19); MS (*m/z*) 391 (40%), (M^+^), 361 (35%), 243 (50%), 155 (100%; loss of naphthoyl fragment), 148 (60%), 136 (65%), 127 (55%; loss of naphthoyl fragment – CO).

*5-(3-Nitrophenyl)-4-amino-1,2,4-triazole-3-ylnaphthalene-1-carbothioate* (**6b**). 5-(3-Nitrophenyl)-1,3,4-oxadiazole-2-ylnaphthalene-1-carbothioate (4.5 g; 12 mmol), NH_2_-NH_2_ (6.20 g; 124 mmol) were reacted according to the general procedure. Color light yellow; yield: 83% (3.80 g); recrystallization from EtOH; m.p. 155-157 °C; Molecular formula C_19_H_13_O_3_N_5_S; Mol. Wt. 191. FTIR (KBr, cm^-1^): 3202 (N-H), 1566 (C=C)_Ar_, 1655 (C=O), 1519 (C=N); ^1^H-NMR (DMSO-d_6_, δ, ppm): 7.60 (dd, 1H, *J* = 3.1, 4.2 Hz, ArH-1), 7.77 (dd, 1H, *J* = 3.2, 4.1 Hz, Ar H-2), 7.04 (dd, 1H, *J* = 2.5, 6.6Hz, A H-3), 7.45 (dd, 1H, *J* = 4.2, 3.1 Hz, Ar H-4), 7.88 (m, 1H, Ar H-1´), 7.79 (m, 1H, Ar H-2´ ), 7.62 (m, 1H, Ar H-3´ ), 8.25 (m, 1H, Ar H-4´ ), 7.21 (m, 1H, Ar H-5´ ), 3.95 (s, 2H, NH_2_); ^13^C-NMR (DMSO-d_6_, δ, ppm): 128.40 (C-1), 127.41 (C-2), 155.50 (C-3), 116.62 (C-4), 118.62 (C-5), 127.42 (C-6), 162.61 (C-7), 174.65 (C-8), 180.37 (C-9), 136.23(C-10), 129.12 (C-11),122.31 (C-12), 125.71(C-13), 128.81 (C-14), 127.50 (C-15), 130.64 (C-16), 132.80 (C-17), 126.21 (C-18), 129.44 (C-19); MS (*m/z*) 391 (40%), (M^+^), 361 (35%), 243 (50%), 155 (100%; loss of naphthoyl fragment) 148 (60%), 136 (65%), 127 (55%; loss of naphthoyl fragment - CO).

*5-Phenyl-4-amino-1,2,4-oxadiazole-3-ylbenzothioate* (**6c**). 5-Phenyl-1,3,4-oxadiazole-2-yl benzo-thioate (4.0 g; 14 mmol), NH_2_-NH_2_ (5.60 g; 113 mmol) were reacted according to the general procedure. Color red; yield: 78% (3.10 g); recrystallization from EtOH; m.p. 214-215 °C; Molecular formula C_15_H_12_ON_4_S; Mol. Wt. 296. FTIR (KBr, cm^-1^): 3212 (N-H), 1574 (C=C)_Ar_, 1664 (C=O), 1525 (C=N); ^1^H-NMR (DMSO-d_6_, δ, ppm): 7.48 (m, 1H, ArH-1) 732 (m, 1H, Ar H-2), 7.22 (m, 1H, A H-3) 7.97 (m, 1H, Ar H-4), 7.65 (dd, 1H, *J* = 3.1, 6.7 Hz, Ar H-1´ ), 7.59 (dd, 1H, *J* = 3.2, 4.4 Hz, Ar H-2´), 7.69 (dd, 1H, *J* = 2.5, 4.7 Hz, Ar H-3´ ), 7.90 (dd, 1H, *J* = 3.1, 6.8 Hz, Ar H-4´ ), 7.45 (dd, 1H, *J* = 2.8, 6.5 Hz, Ar H-5´ ), 4.12 (s, 2H, NH_2_); ^13^C-NMR (DMSO-d_6_, δ, ppm): 130.05 (C-1), 126.41 (C-2), 116.24 (C-3), 115.24 (C-4), 116.37 (C-5), 126.21 (C-6), 166.04 (C-7), 176.23 (C-8), 184.32 (C-9), 134.05 (C-10), 130.73 (C-11),126.32 (C-12), 125.61 (C-13), 126.67 (C-14), 130.73 (C-15); MS (*m/z*) 296 (42%) (M^+^), 266 (35%), 193 (50%), 103 (62%),105 (100%; loss of acylium ion fragment), 77 (52%; loss of phenyl cation fragment).

*5-Phenethyl-4-amino-1,2,4-triiazole-3-yloctanethioate* (**6d**). 5-Phenethyl-1,3,4-oxadiazole-2-yl octane-thioate (6.0 g; 18 mmol), NH_2_-NH_2_ (8.20 g; 165 mmol) were reacted according to the general procedure. Color orange; yield: 74% (4.40 g); recrystallization from EtOH; m.p. 179-180 °C; Molecular formula C_18_H_26_ON_4_S; Mol. Wt. 346. FTIR (KBr, cm^-1^): 3210 (N-H), 1520 (C=C)_Ar_, 1660 (C=O), 1582 (C=N); ^1^H-NMR (DMSO-d_6_, δ, ppm):; ^1^HNMR (DMSO-d_6_) δ (ppm): 7.12 (m, 1H, ArH-1), 7.22 (dd, 1H, *J* = 2.4, 6.7 Hz, ArH-2), 7.08 (m, 1H, ArH-3), 7.22 (dd, 1H, *J* = 2.4, 6.7 Hz, ArH-2), 4.33 (s, 2H, NH_2_), 2.88, 3.2, 1.5 (t, 4H, t, 2H, m, 10 H other protons) 2.50, 3.2, 1.52 (t, 2H, m, 10H, other protons); ^13^C-NMR (DMSO-d_6_, δ, ppm): 28.36 (C-1), 30.18 (C-2), 127.21 (C-3), 126.33 (C-4), 127.41 (C-5), 116.24 (C-6), 118.44 (C-7), 116.26 (C-8), 166.23 (C-9), 175.90(C-10), 182.64 (C-11),45.46 (C-12), 33.67(C-13), 26.34 (C-14), 25.80 (C-15), 20.21 (C-16), 18.44 (C-17), 22.37 (C-18); MS (*m/z*) 346 (40%) (M^+^), 316 (36%), 215 (50%), 131 (60%), 127 (100%), 99 (55%).

*5-Phenethyl-4-amino-1,2,4-triazole-3-ylbenzothioate* (**6e**). 5-Phenethyl-1,3,4-oxadiazole-2-yl benzo-thioate (5.0 g; 16 mmol), NH_2_-NH_2_ (7.20 g; 144 mmol) were reacted according to the general procedure. Color light yellow; yield: 80% (4.00 g); recrystallization from EtOH; m.p. 158-159 °C; Molecular formula C_17_H_16_ON_4_S; Mol. Wt. 324. FTIR (KBr, cm^-1^): 3195 (N-H), 1509 (C=C)_Ar_, 1673 (C=O), 1567 (C=N); ^1^H-NMR (DMSO-d_6_, δ, ppm): 7.14 (m, 1H, ArH-1), 7.47 (dd, 1H, *J* = 2.1, 7.2 Hz, Ar H-2), 7.12 (m, 1H, ArH-3), 7.67 (m, 1H, Ar H-4), 7.97 dd, 1H, *J* = 2.4, 6.5 Hz, ArH-1´), 7.58 (dd, 1H *J* = 3.1, 4.5 Hz, Ar-H-2´), 7.77 (m, 1H ArH-3´), 7.67 (m, 1H ArH-4´) 4.16 (s, 2H, NH_2_), 2.90 (t, 4H other protons); ^13^C-NMR (DMSO-d_6_, δ, ppm): 30.33 (C-1), 32.71 (C-2), 130.43 (C-3), 118.23 (C-4), 117.23 (C-5), 115.48 (C-6), 118.23 (C-7), 125.63 (C-8), 159.21 (C-9), 170.62 (C-10), 183.47 (C-11),134.24 (C-12), 128.40 (C-13), 129.65 (C-14), 126.31 (C-15), 130.52 (C-16), 127.43 (C-17); MS (*m/z*) 324 (45%) (M^+^), 294 (35%), 193 (50%), 131 (62%), 105 (100%; loss of acylium ion fragment), 77 (52%; loss of phenyl cation fragment).

*5-(3-Nitrophenyl)-4-amino-1,2,4-triazole-3-ylheptanethioate* (**6f**). 5-(3-Nitrophenyl)-1,3,4-oxadiazole-2-ylheptanethioate (3.0g; 8 mmol), NH_2_-NH_2_ (5.10 g; 103 mmol) were reacted according to the general procedure. Color golden; yield: 76% (2.30 g); recrystallization from EtOH; m.p. 148-150 °C; Molecular formula C_15_H_19_O_3_N_5_S; Mol. Wt. 349. FTIR (KBr, cm^-1^): 3140 (N-H), 1570 (C=C)_Ar_, 1667 (C=O), 1514 (C=N); ^1^H-NMR (DMSO-d_6_, δ, ppm): 7.47 (dd, 1H, *J* = 3.2, 6.2 Hz, ArH-1), 7.27 (dd, 1H, *J* = 2.1, 7.2 Hz, Ar H-2), 6.97 (dd, 1H, *J* = 3.2, 7.3 Hz, Ar H-3), 7.47 (dd, 1H, *J* = 3.4, 6.2 Hz, Ar H-4), 3.94 (s, 2H, NH_2_), 2.50, 3.2, 1.52 (t, 2H m, 10H, other protons); ^13^C-NMR (DMSO-d_6_, δ, ppm): 130.06 (C-1), 127.25 (C-2), 152.44 (C-3), 127.25 (C-4), 126.31 (C-5), 117.21 (C-6), 161.37 (C-7), 170.68 (C-8), 182.04 (C-9), 54.34 (C-10), 44.31 (C-11),33.39 (C-12), 24.36 (C-13), 25.62 (C-14), 20.22 (C-15); MS (*m/z*) 349 (40%) (M^+^), 319 (37%), 201 (50%), 148 (60%),113 (100%), 85 (55%).

*5-(4-Methoxybenzyl)-4-amino-1,2,4-triazole-3-ylnaphthalene-1-carbothioate* (**6g**). 5-(4-Methoxy-benzyl)-1,3,4-oxadiazole-2-yl naphthalene-1-carbothioate (5.0 g; 13 mmol), NH_2_-NH_2_ (8.20 g; 165 mmol) were reacted according to the general procedure. Color reddish brown; yield: 82% (4.10 g); recrystallization from EtOH; m.p. 166-167 °C; Molecular formula C_21_H_18_O_2_N_4_S; Mol. Wt. 390. FTIR (KBr, cm^-1^): 3204 (N-H), 1575 (C=C)Ar, 1684 (C=O), 1518 (C=N); ^1^H-NMR (DMSO-d_6_, δ, ppm): 6.50 (m, 1H, ArH-1), 6.87 (m, 1H, Ar H-2), 6.50 (m, 1H, A H-3), 6.87 (m, 1H, Ar H-4), 7.56 (dd, 1H, *J* = 2.4, 6.2 Hz, Ar H-1´ ), 7.42 (dd, 1H, *J* = 3.5, 6.5 Hz, Ar H-2´ ), 7.73 (m, 1H, Ar H-3´ ), 7.46 (m, 1H, Ar H-4´ ), 7.55 (m, 1H, Ar H-5´ ), 3.83 (s, 2H, NH_2_), 2.50, (s, 2H, methylene protons) 3.56 (s, 3H, methyl protons); ^13^C-NMR (DMSO-d_6_, δ, ppm): 30.60 (C-1), 55.90 (C-2), 130.02 (C-3), 127.05 (C-4), 116.65 (C-5), 158.03 (C-6), 166.04 (C-7), 175.10 (C-8), 180.01 (C-9), 116.65 (C-10), 127.05 (C-11),134.50 (C-12), 127.12 (C-13), 125.62 (C-14), 120.50 (C-15), 119.42 (C-16), 129.31 (C-17), 131.61 (C-18), 130.71 (C-19), 123.63 (C-20); MS (*m/z*) 390 (40%) (M^+^), 360 (37%), 243 (50%), 147 (60%), 155 (100%; loss of naphthoyl fragment) 148 (60%), 136 (65%), 127 (55%; loss of naphthoyl fragment - CO) 121 (65%; loss of tropylium ion).

*5-Phenethyl-4-amino-1,2,4-triazole-3-ylnonanethioate* (**6h**). 5-Phenethyl-1,3,4-oxadiazole-2-yl nonane-thioate (4.0 g; 12 mmol), NH_2_-NH_2_ (5.60 g; 113 mmol) were reacted according to the general procedure. Color yellow; yield: 78% (3.10 g); recrystallization from EtOH; m.p. 138-139 °C; Molecular formula C_17_H_24_ON_4_S; Mol. Wt. 332. FTIR (KBr, cm^-1^): 3260 (N-H), 1512 (C=C)_Ar_, 1652 (C=O), 1590 (C=N); ^1^H-NMR (DMSO-d_6_, δ, ppm): 6.52.(m, 1H, ArH-1), 6.77 (m, 1H, Ar H-2), 7.14 (m, 1H, A H-3), 7.02 (m, 1H, Ar H-4), 4.45 (s, 2H, NH_2_), 2.50, 3.2, 1.60 (t, 2H, m, 12H other protons); ^13^C-NMR (DMSO-d_6_, δ, ppm): 130.46 (C-1), 125.34 (C-2), 116.73 (C-3), 118.62 (C-4), 116.73 (C-5), 125.23 (C-6), 159.68 (C-7), 168.77 (C-8), 184.66 (C-9), 53.62 (C-10), 44.02 (C-11),34.71 (C-12), 29.01 (C-13), 25.72 (C-14), 25.69 (C-15), 20.02 (C-16), 18.33 (C-17). MS (*m/z*) 332 (40%) (M^+^), 302 (37%), 229 (52%), 141 (100%), 113 (50%), 103 (58%)

*5-(3-Nitrophenyl)-4-amino-1,2,4-triazole-3-ylbenzothioate* (**6i**). 5-(3-Nitrophenyl)-1,3,4-oxadiazole-2-ylbenzothioate (3.0 g; 9 mmol), NH_2_-NH_2_ (5.10 g; 103 mmol) were reacted according to the general procedure. Color brown; yield: 66% (2.10 g); recrystallization from EtOH; m.p. 138-139 °C; Molecular formula C_15_H_11_O_3_N_5_S; Mol. Wt. 341. FTIR (KBr, cm^-1^): 3318 (N-H), 1510 (C=C)_Ar_, 1625 (C=O), 1587 (C=N); ^1^H-NMR (DMSO-d_6_, δ, ppm): 7.12 (dd, 1H, *J* = 3.2, 6.4 Hz, ArH-1),7.27 (dd, 1H, *J* = 3.2, 7.4 Hz, Ar H-2), 7.12 (dd, 1H, *J* = 3.2, 6.4 Hz, A H-3), 7.77 (m, 1H, Ar H-4), 7.67 (m, 1H, Ar H-1´), 7.85 (m, 1H, Ar H-2´), 7.58 (m, 1H, Ar H-3´), 7.82 (m, 1H, Ar H-4´), 7.25 (m, 1H, Ar H-5´), 4.26 (s, 2H, NH_2_); ^13^C-NMR (DMSO-d_6_, δ, ppm): 133.06 (C-1), 127.45 (C-2), 153.44 (C-3), 125.21 (C-4), 127.40 (C-5), 117.67 (C-6), 163.23 (C-7), 177.45 (C-8), 181.08 (C-9), 136.38 (C-10), 127.21 (C-11),116.90 (C-12), 126.64 (C-13), 128.03 (C-14), 125.64 (C-15); MS (*m/z*) 341 (42%) (M^+^), 311 (35%), 193 (50%), 148 (60%), 105 (100%; loss of acylium ion fragment), 77 (52%; loss of phenyl cation fragment).

### 3.8. Antifungal evaluation

The synthesized compounds were tested by agar tube dilution method [17] for their *in vitro* fungicidal activity. Terbinafine (200 μg/mL) was used as positive control. All experiments were done in three replicates. Four fungal strains: *Aspergillus flavus, Mucor species, Aspergillus niger* and *Aspergillus fumigatus* were used. All fungal strains were grown on 6.5% SDA (Sabouraud dextrose agar, pH 5.7) at 28 °C and preserved at 4 °C in a refrigerator. One hundred mm slants with sterilized SDA were prepared by adding each compound in 200 μg/mL concentration. Terbinafine (200 μg/mL) was used as standard drug while DMSO was used as negative control. Each slant was inoculated with 4 mm piece of respective fungal strain and incubated at 28 °C for 7-10 days. Fungal growth was compared with negative control to get the %age inhibition. 

## 4. Conclusions 

Nine 5-substitued-1,3,4-oxadiazole-2-thioesters derivatives **5a-i** containing different groups and obtained *via* 5-substituted-1,3,4-oxadiazole-2-thiols using potassium hydroxide and acid chloride were subjected to reactions with hydrazine hydrate to furnish nine new 5-substituted-4-amino-1,2,4-triazole-3-thioesters **6a-i**.Their structures were confirmed by infrared, ^1^H- and ^13^C -NMR and mass spectrometric analysis. In the *in vitro* antifungal bioassay, compounds **6a, 6b, 6d** and **6g** were found to have significant antifungal activities against the four tested fungal strains: *Aspergillus flavus, Mucor species, Aspergillu niger* and *Aspergillu. fumigatus* Structure activity relationship comparison shows that the presence of naphthyl group at 3-position of the triazole-3-thioester enhances the activity of the compounds, probably because a similar group is also present in the structure of the reference compound terbinafine.

## Figures and Tables

**Table 1:** ^1^H-NMR spectral data of 5-substitued-4-amino-1,2,4-triazole-3-thioesters **6a-i**.

Comp.	Protons [^1^H-δ(ppm), Multiplicity, J(Hz)]										Other protons
	Ar–H									NH_2_	
	H-1	H-2	H-3	H-4	H-1′	H-2′	H-3′	H-4′	H-5′		
**6a**	7.72 1H,dd 1.2,4.6	8.03 1H,dd 2.5,7.2	8.01 1H,dd 2.4,7.1	7.73 1H,dd 2.3,4.4	7.76 1H,m	7.42 1H,m	7.90 1H,m	7.88 1H,m	7.55 1H,m	4.32 2H,s	–
**6b**	7.60 1H,dd 3.1,4.2	7.77 1H,dd 3.2,4.1	7.04 1H,dd 2.5,6.6	7.45 1H,dd 4.2,3.1	7.88 1H,m	7.79 1H,m	7.62 1H,m	8.25 1H,m	7.21 1H,m	3.95 2H,s	–
**6c**	7.48 1H,m	7.32 1H,m	7.22 1H,m	7.97 1H,m	7.65 1H,dd 3.1,6.7	7.591H,dd1H,m	7.69 1H,dd 2.5,4.7	7.98 1H,dd 3.1,6.8	7.45 1H,dd 2.8,6.5	4.122H,s	–
**6d**	7.12 1H,m	7.22 1H,dd 2.4,6.7	7.08 1H,m	7.22 1H,dd 2.4,6.7	–	–	–	–	–	4.33 2H,s	2.88 4H,t 3.2 2H,t 2.1,1.5 10H,m
**6e**	7.14 1H,m	7.47 1H,m	7.12 1H,m	7.67 1H,m	7.97 1H,dd 2.4,6.5	7.58 1H,dd 3.1,4.5	7.77 1H,m	7.67 1H,m	–	4.16 2H,s	2.90 4H,t 2.8
**6f**	7.47 1H,dd 3.2,6.2	7.27 1H,dd 2.1,7.2	6.97 1H,dd 3.2,7.3	7.47 1H,dd 3.4,6.2	–	–	–	–	–	3.94 2H,s	2.50 2H,t 3.2,1.52 10H,m
**6g**	6.50 1H,m	6.87 1H,m	6.50 1H,m	6.87 1H,m	7.76 1H,dd 2.4,6.2	7.42 1H,dd 3.5,6.5	7.73 1H,m	7.46 1H,m	7.55 1H,m	3.83 2H,s	2.50 2H,s 3.56 3H,s
**6h**	6.52 1H,m	6.77 1H,m	7.14 1H,m	7.02 1H,m	–	–	–	–	–	4.45 2H,s	2.5 2H,t 3.2,1.60 12H,m
**6i**	7.12 1H,dd 2.3,6.4	7.27 1H,dd 3.2,7.4	7.12 1H,dd 2.3,6.4	7.77 1H,m	7.67 1H,m	7.85 1H,m	7.58 1H,m	7.82 1H,m	7.25 1H,m	4.26 2H,s	–

**Table 2:** ^13^C-NMR spectral data of 5-substitued-4-amino-1,2,4-triazole-3-thioesters **6a-i**.

Carbon No.	^13^C-δ(ppm), Compounds								
	6a	6b	6c	6d	6e	6f	6g	6h	6i
1	129.41	128.40	130.05	28.36	30.33	130.06	30.60	130.46	133.06
2	128.40	127.41	126.41	30.18	32.71	127.25	55.90	125.34	127.45
3	116.52	155.50	116.24	127.21	130.43	152.44	130.02	116.73	153.44
4	115.52	116.62	115.24	126.33	118.23	127.25	127.05	118.62	125.21
5	116.31	118.62	116.37	127.41	117.23	126.31	116.65	116.73	127.40
6	130.43	127.42	126.21	116.24	115.48	117.21	158.03	125.23	117.67
7	160.42	162.61	166.04	118.44	118.23	161.37	166.04	159.68	163.23
8	171.40	174.65	176.23	116.26	125.63	170.68	175.10	168.77	177.45
9	182.31	180.37	184.32	166.23	159.21	182.04	180.01	184.66	181.08
10	135.61	136.23	134.05	175.90	170.62	54.34	116.65	53.62	136.38
11	127.12	129.12	130.73	182.64	183.47	44.31	127.05	44.02	127.21
12	120.43	122.31	126.32	45.46	134.24	33.39	134.50	34.71	116.90
13	122.41	125.71	125.61	33.67	128.40	24.36	127.12	29.01	126.64
14	125.32	128.81	126.67	26.34	129.65	25.62	125.62	25.72	128.03
15	126.32	127.50	130.73	25.80	126.31	20.22	120.50	25.69	125.64
16	128.43	130.64	–	20.21	130.52	–	119.42	20.02	–
17	129.42	132.80	–	18.44	127.43	–	129.31	18.33	–
18	130.21	126.21	–	22.37	–	–	131.61	–	–
19	129.66	129.44	–	–	–	–	130.71	–	–
20	–	–	–	–	–	–	123.63	–	–

**Table 3:** Antifungal activity of 5-substituted-4-amino-1,2,4-triazole-3-thioesters.

Sample No.	Structure of 5-substituted-4-amino-1,2,4-triazole-thioester	Fungal strains/ Inhibition (%)			
		*Aspergillus flavus*	*Mucor species*	*Aspergillus niger*	*Aspergillus flavus*
**6a**	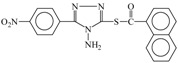	96.22	87.66	74.79	97.20
**6b**	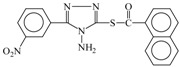	84.12	100.00	81.20	100.00
**6c**	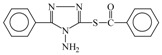	69.00	80.90	75.20	87.00
**6d**	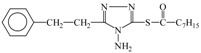	81.00	78.36	59.19	93.60
**6e**	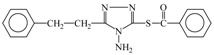	71.00	45.89	69.99	73.00
**6f**	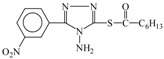	27.00	15.58	24.39	75.00
**6g**	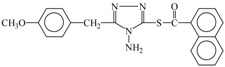	100.00	88.64	98.20	88.00
**6h**	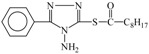	54.00	21.22	20.00	52.00
**6i**	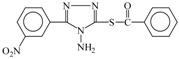	50.00	19.48	51.99	88.00
**Terbinafine standard drug**	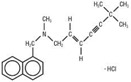	100.00	90.00	110.80	98.40
